# Unveiling the hidden threats: a review of pathogen diversity and public health risks from bats, rodents, and non-human primates in Zambia (1990–2022)

**DOI:** 10.3389/fpubh.2024.1471452

**Published:** 2024-11-20

**Authors:** Samuel Munalula Munjita, Benjamin Mubemba, Katendi Changula, John Tembo, Raymond Hamoonga, Matthew Bates, Simbarashe Chitanga, Sody Munsaka, Edgar Simulundu

**Affiliations:** ^1^Department of Biomedical Sciences, School of Health Sciences, University of Zambia, Lusaka, Zambia; ^2^Department of Wildlife Sciences, School of Natural Resources, Copperbelt University, Kitwe, Zambia; ^3^Department of Paraclinical Studies, School of Veterinary Medicine, University of Zambia, Lusaka, Zambia; ^4^HerpeZ, University Teaching Hospital, Lusaka, Zambia; ^5^Zambia National Public Health Institute, Lusaka, Zambia; ^6^School of Natural Sciences, University of Lincoln, Lincoln, Lincolnshire, United Kingdom; ^7^Department of Preclinical Studies, School of Veterinary Medicine, University of Namibia, Windhoek, Namibia; ^8^Macha Research Trust, Choma, Zambia

**Keywords:** Zambia, bats, rodents, non-human primates, pathogens, public health, surveillance, zoonoses

## Abstract

**Background:**

Infectious disease agents of animal origin, which can cause mild to severe illnesses in humans, are increasingly spilling over into human populations. Southern Africa, particularly Zambia as a regional transport hub, has experienced notable outbreaks of zoonotic pathogens in recent years. This context underscores the importance of research, as numerous studies over the past 33 years have reported various infectious agents with differing zoonotic potential from bats, rodents, and non-human primates (NHPs) in Zambia. However, the data remained unaggregated, hampering comprehensive and organized understanding of these threats.

**Methods:**

A review spanning January 1990 to December 2022 synthesised data from selected studies conducted in bats, rodents, and NHPs across 14 of Zambia’s 116 districts.

**Results:**

Among the reported pathogens, viruses predominated (62%, 31/50), followed by parasites (20%, 10/50)), and bacteria (18%, 9/50). Notable pathogens included Ebola virus, Marburg virus, Hantavirus, Zika virus, Human parainfluenza virus-3, *Anaplasma phagocytophilum*, *Borrelia faini*, *Coxiella burnetii*, *Trypanosoma brucei rhodesiense*, *Calodium hepaticum*, and *Trichinella spiralis*. Most identified infectious agents came from short term cross-sectional investigations, thus, the temporal dynamics related to abundance and likelihood of outbreaks remain unknown.

**Conclusion:**

The findings starkly illuminate significant zoonotic public health threats amidst glaring under-surveillance of zoonoses in humans in Zambia. This critical gap calls urgently for enhanced active, passive and syndromic surveillance activities to identify new diseases and provide evidence-based measures to safeguard public health from emerging infectious risks in Zambia and the Southern African sub-region, considering the country’s position as a regional transport hub.

## 1 Introduction

In 2008, a mysterious outbreak of a haemorrhagic fever virus disease occurred in Zambia and South Africa killing 80% of the infected people (1). The origin and host of the virus later identified as Lujo virus remains unknown to date. This and several other examples continuously indicate that infectious agents (zoonoses) originating from domestic animals and wildlife present profound threats to global public health and trade (2). These diseases, often unpredictable and challenging to treat, have been increasingly linked to wildlife (3). Since 1940, approximately 60% of human infectious diseases have emerged from animal reservoirs, with more expected to cross into human populations by 2070 or earlier (4–6). These zoonotic pathogens cause a billion of human illnesses and millions of deaths annually (4, 7), transmitted through various means including consumption of infected food (fruits, meats, vegetables, and date palm sap), aerosols, secretions (saliva), excreta (urine and faeces), and handling animals and their products (8–11). The magnitude and complexity of these transmission pathways underscore the urgent need for comprehensive surveillance and robust public health interventions to mitigate these escalating risks.

Over the past seven decades, certain animals have been identified as key carriers of zoonotic pathogens. Bats, rodents, and non-human primates (NHPs) are particularly significant due to their widespread presence and close interactions with humans (12–15). Bio-diversity loss related to agriculture, environmental changes, and other commercial activities have been linked to increased contact between humans and wildlife and cross-species transmission of pathogens (2–4). Bats and rodents, in particular, often live commensally or semi-commensally in human dwellings (8, 16). Bats, capable of long-distance flight, spread viruses such as Nipah (17) and Marburg (18), and are implicated in the origins of Ebola virus (19) and severe acute respiratory syndrome (SARS) coronaviruses −1 and 2 (SARS-CoV-1 and SARS-CoV-2) (20, 21). On the other hand, Lassa virus (LASV) and *Yersinia pestis* are among the deadly pathogens directly linked to rodents (22, 23) while Lujo virus (LUJV) is suspected to have a rodent reservoir (24). The unpredictable emergence of LUJV, a hemorrhagic fever virus first identified in 2008, underscores the critical importance of vigilance and review of information, as its host and transmission dynamics remain unknown. Meanwhile, NHPs, due to their genetic similarities to humans and increasing habitat overlap due to habitat losses linked to agriculture, are notably implicated in the zoonotic origin of HIV and may serve as potential intermediaries of Ebola virus, particularly during an outbreak (25–28). Addressing these complex interactions demands sustained monitoring and proactive public health strategies to mitigate the evolving risks posed by zoonotic pathogens.

Given the substantial evidence of zoonotic threats posed by these animals (12–15), this study reviewed literature from January 1990 to December 2022 to examine the epidemiology and public health implications of pathogens in bats, rodents, and NHPs in Zambia. In the light of these threats, weak health systems with little capacity for multi-pathogen laboratory diagnosis (29), aggregated data may help identify hotspots which may guide presumptive clinical diagnosis in specific regions, and enhance our ability to respond to outbreaks effectively. The organized information may also inform policymakers, public health practitioners, clinicians, researchers, and financial stakeholders, providing critical insights for diagnosis, treatment, future actions and research in order to safeguard human and animal health.

## 2 Methods

### 2.1 Information sources and search strategies

A comprehensive data search was conducted across three electronic databases including (PubMed, Google Scholar, and CiNii Articles Incorporated Database) to identify articles and accompanying data reporting pathogens in bats, rodents, and NHPs. The search was restricted to studies published between January 1990 and December 2022. The period was selected in order to provide a comprehensive and nuanced understanding of the research trends and distribution of pathogens in the light of continuous changes in globalisation, pandemics, research tools national priorities, data availability, and demographic changes. Eligible original studies were identified using the following search terms with the help of Boolean operators (AND, OR): ((Virus) AND (Zambia)) AND (bat), ((Virus) AND (Zambia)) AND (rodent), ((Virus) AND (Zambia)) AND (non-human primates), ((Virus) AND (Zambia)) AND (baboons), ((Virus) AND (Zambia)) AND (Monkeys), ((Bacteria) AND (Zambia)) AND (bats), ((Bacteria) AND (Zambia)) AND (rodent), ((Bacteria) AND (Zambia)) AND (non-human primates), ((Bacteria) AND (Zambia)) AND (baboons), ((Bacteria) AND (Zambia)) AND (Monkeys), ((Protozoa) AND (Zambia)) AND (bats), ((Protozoa) AND (Zambia)) AND (rodent), ((Protozoa) AND (Zambia)) AND (non-human primates), ((Protozoa) AND (Zambia)) AND (baboons), ((Protozoa) AND (Zambia)) AND (Monkeys), ((Zoonoses) AND (Zambia)) AND (bats), ((Zoonoses) AND (Zambia)) AND (rodent), ((Zoonoses) AND (Zambia)) AND (non-human primates), ((Zoonoses) AND (Zambia)) AND (baboons), ((Zoonoses) AND (Zambia)) AND (Monkeys), ((Pathogen) AND (Zambia)) AND (bats), ((Pathogen) AND (Zambia)) AND (rodent), ((Pathogen) AND (Zambia)) AND (non-human primates), ((Pathogen) AND (Zambia)) AND (baboons), and ((Pathogen) AND (Zambia)) AND (Monkeys). The search strategy also included ((bats OR rodents OR “non-human primates” OR baboons OR monkeys) AND (bacteria OR protozoa OR zoonoses) AND (Zambia) AND (prevalence OR seroprevalence OR wildlife OR national park)) Following identification of some articles which specified certain pathogens, search terms were extended to include some names of identified pathogens as follows: ((bats OR rodents OR “non-human primates” OR baboons OR monkeys) AND (bacteria OR protozoa OR zoonoses OR Trypanosomes OR Giardia OR Cryptosporidium OR Coxiella OR Borrelia OR Paramyxovirus OR Leptospira OR Filovirus OR “Marburg virus” OR “Hepatitis virus” OR Rotavirus OR Arenavirus OR Rickettsia OR Babesia OR Anaplasma) AND (Zambia)).

### 2.2 Inclusion criteria

Selected studies focused on rodents, bats, or NHPs in Zambia, reporting on sample size and type, diagnostic methods, and pathogen type. Studies had to be peer-reviewed, in English, fully accessible, conducted in Zambia, and published from January 1, 1990, to December 31, 2022. Experimental studies that combined both surveillance and experimentation were also included.

### 2.3 Exclusion criteria

Studies were excluded if they lacked detailed methodological descriptions, did not report prevalence data, were not peer-reviewed, or were not written in English. Journal articles published outside the predetermined review period, and duplicates as well as non-original research were excluded from the review.

### 2.4 Data extraction and management

Data were independently extracted for each pathogen or potential pathogen by two reviewers (SMM and BM) using a standardised form for this review. It included animal species (rodent, bat, NHP), sample size, type, diagnostic methods, pathogen type, positives, and genetic matches via BLAST. Other data covered study location, design, publication year, authors, and journal. Discrepancies were resolved through discussion or a third reviewer who was available. Data were managed in Microsoft Office Excel 2018, ensuring integrity with backups. Zotero (Version 5.0.96.3) stored study details including titles, abstracts, authors, years, journals, and extracted PDFs where available, facilitating comprehensive information retrieval and management. The quality of the studies was evaluated using the JBI’s critical appraisal tools for prevalence and incidence studies to assess the trustworthiness, relevance, and results of the studies (30).

### 2.5 Data synthesis and analysis

The prevalence rate of pathogens in selected animal species was directly extracted from the reviewed articles. For studies reporting data on multiple animal species, the prevalence was recalculated separately for each species of interest to ensure accurate representation (31–33). In instances where data from multiple studies were available, the cumulative prevalence of certain pathogens was determined by combining data from these studies. The public health risk associated with each reported pathogen was assessed based on existing evidence from the included articles and relevant literature demonstrating the pathogen’s potential to cause disease.

### 2.6 Data presentation

Descriptive data were summarised in tables showing the number of samples analysed, number of positive cases, and prevalence rates. Maps were created to show the distribution of sample collection sites across Zambia and reported highly infectious agents. A detailed summary of findings was presented, including a discussion and implications for future research.

### 2.7 Ethical considerations

All included studies were evaluated for adherence to ethical standards for animal research. This included reviewing whether the original studies obtained appropriate ethical approvals and consent for animal use.

## 3 Results

### 3.1 Literature search

The literature search identified 37 eligible articles. The number represents the actual number of surveillance studies conducted specifically on bats, rodents, and NHPs in Zambia from 1990 to 2022.

### 3.2 Publication trends

A total of thirty-seven original research articles were included in the analysis, covering the period January 1, 1990 to December 31, 2022. The search did not yield any articles within the investigated databases for the period 1990 to 2009. The highest publication frequency was observed in the years 2018 and 2019 (n = 10). When categorised by host type, 41%% (14/39) of the articles represented studies focused on bats (34–47), 30.8% (12/39) on rodents (23, 31, 48–56), and 25.6% (10/39) on NHPs (32, 57–65). One article (2.6%; 1/39) reported on both NHPs and rodents (33). In terms of pathogens detected, 66.7% (26/39) of the articles investigated viruses exclusively (31–34, 37–40, 43–47, 50–52, 55, 58–64), 17.9% (7/39) focused solely on bacteria (23, 35, 36, 41, 48, 53, 54), and 5.1% (2/39) protozoa alone (42). The remaining 10.3% (4/39) of the articles addressed mixed infections involving bacteria and viruses (49), helminths and viruses (56), as well as bacteria and protozoa (57).

### 3.3 Study sites, sampling approaches, and sample types

**Figure 1:**
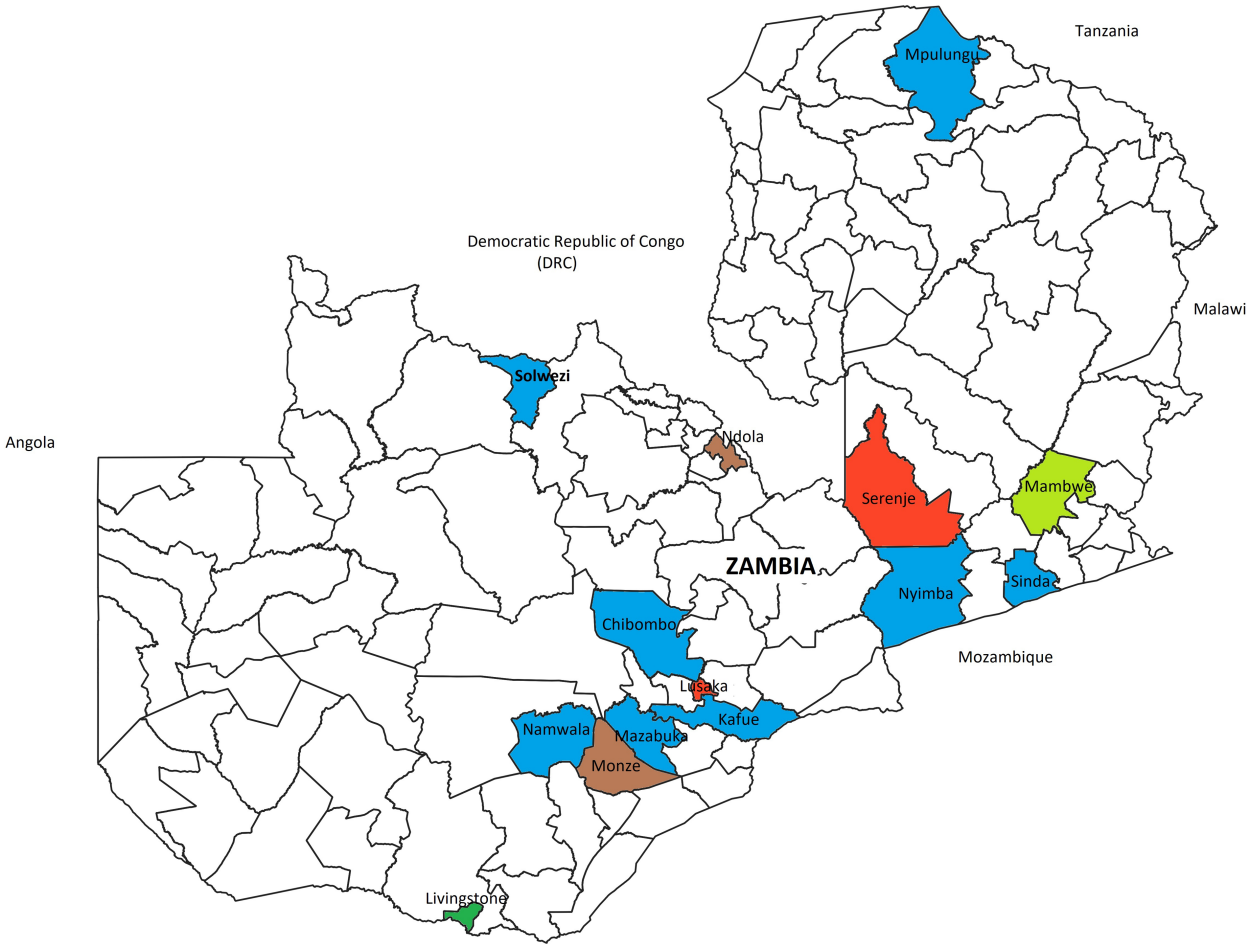
Sampling sites, derived from reviewed articles, for pathogens detected in bats, rodents, and NHPs across Zambia. Districts in which only rodents were sampled (Blue), bats only (Brown), rodents and NHPs (lime green), rodents and bats (red) and all animal types (Green).

Samples were collected from animals in study sites across 14 out of the 116 districts in Zambia (Figure 1). Reasons for selecting the reported study sites were not indicated. All the articles reported use of cross-sectional study designs. Short-term cross-sectional studies accounted for 94.9% (37/39) of all reported articles compared to 5.4% (2/37) of long term cross-sectional studies (38, 59). Almost all articles (97.4%, 38/39) had spleen, liver, kidneys, and blood as samples of choice with the exception of one study (2.6%, 1/39) in rodents that extended its investigations to semen (56).

### 3.4 Diversity and geographical distribution of reported bat, rodent, and NHP species

Thirteen species of bats were reported within the study areas in Livingstone, Lusaka, Monze, Ndola and Serenje districts (34–47). The reported species of bats included *Hipposideros gigas*, *Hipposideros vittatus*, *Miniopterus schreibersii*, *Rousettus aegyptiacus*, *Minipteros* sp.*, Myotis* sp., *Rhinolophus simulator*, *Macronycteris vittatus,* and other unknown species in the genera *Rhinolophus* and *Hipposideros* in Lusaka province where most of the investigations took place; *Eidolon helvum* in Ndola and Serenje (Kasanka National Park) districts; *Epomophorus crypturus* in Monze district; and *Nycteris* sp. in Livingstone district (34–47). In terms of common species, *Rousettus aegyptiacus* (23.2%, 719/3100) and *Eidolon helvum* (68.2%, 2114/3100) were the most captured species of bats, accounting for 91.4% (2,833/3100) of all reported bats.

Rodents belonging to 17 genera were identified in sampling sites in Chibombo, Kafue, Livingstone, Lusaka, Mazabuka, Mfuwe, Mpulungu, Namwala, Nyimba, Serenje, Sinda, and Solwezi districts. Reported rodent species included *Acomys subspinosus*, *Aethomys chrysophilus*, *Arvicanthis* sp., *Cricetomys gambianus*, *Gerbilliscus leukogaster*, *Grammomys* sp., *Graphiurus* sp., *Hylomyscus alleni*., *Praomys* sp., *Lemniscomys rosalia*, *Mastomys natalensis*, *Mus minutoides*, *Otomys* sp., *Rattus rattus*, *Saccostomus campestris*, *Pelomys* sp. (23, 31, 33, 48–53, 55). The distribution of rodents was fairly ubiquitous across the districts except for *Mus minutoides* which was only reported in Lusaka district. The most trapped rodent species was *M. natalensis* which accounted for 91.4% (1825/1996) of all captured rodents.

*Chlorocebus* and *Papio* were the only genera of NHPs reported in the reviewed articles (32, 57, 59–61, 64). The genus *Chlorocebus* was represented by *Chlorocebus pygerythrus* (vervet monkey) and *Chlorocebus cynosures*. Meanwhile, kinda yellow baboon (*Papio kindae*), yellow baboons (*Papio cynocephalus*), and Chacma baboons (*Papio ursinus*) comprised NHPs in the genus *Papio*. All species were reported in the sampling sites in Livingstone and Mfuwe districts except in the Kafue National Park where only *Chlorocebus cynosures* were sampled. The most captured NHP was *P. cynocephalus* (28.3%, 339/1198).

### 3.5 Diversity of infectious agents reported in bats, rodents, and NHPs

**Figure 2:**
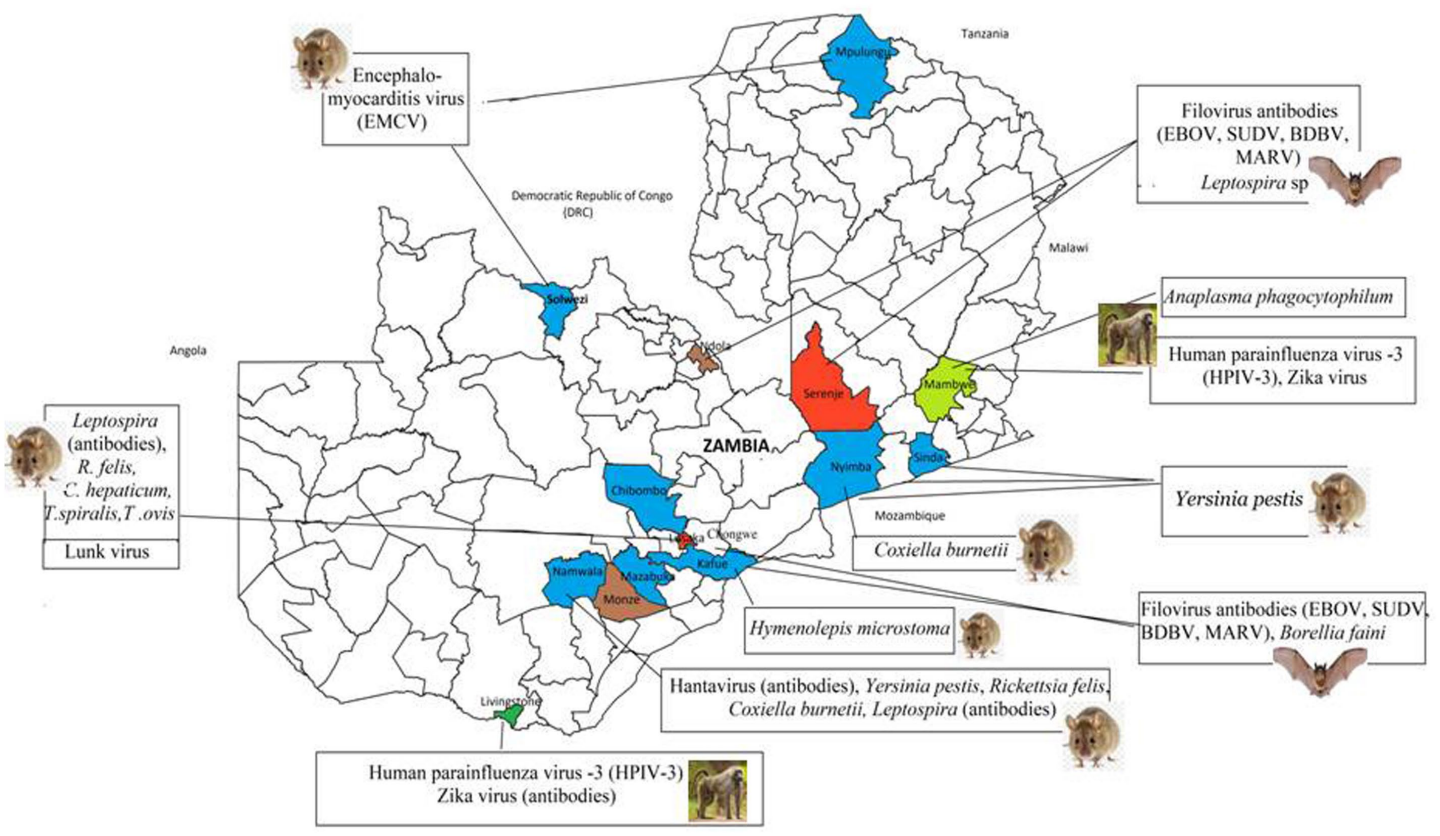
Distribution of high risk infectious agents reported in wildlife in the reviewed articles. Coloured areas represent the location of study sites from the 37 articles.

A combined total of 50 distinct infectious agents were reported in the reviewed articles. Bats and rodents each accounted for 36% (18/50) of all infectious agents whereas NHPs were responsible for 28% (14/50). Viruses were primarily the most frequently reported types of microorganisms accounting for 62% (31/50), mirroring the focus of the research in the reviewed articles. A total of 18% (9/50) of infectious agents were bacteria and the remaining 20% (10/50) were parasites. The array of infectious agents across bats, rodents, and NHPs represented highly infectious pathogens (Figure 2) and those with unknown zoonotic potential (Tables 1–4; Supplementary Table S1).

**Table 1:** Diversity of bacteria, parasites, and viruses reported in the reviewed articles.

Category	Infectious disease agent
Bacteria	*Candidatus Bartonella rousetti* (*Ca. Bartonella rousetti*), *Anaplasma phagocytophilum*, *Rickettsia* sp., *Borrelia faini*, *Leptospira* sp., *Yersinia pestis*, *Rickettsia felis*, *Coxiella burnetii*
Parasites	*Babesia* sp., *Trypanosoma* sp., *T. brucei rhodesiense*, *Hymenolepis microstoma*, *Caenorhabditis inopinata*, *Aonchotheca paranalis*, *Calodium hepaticum*, *Trichinella spiralis*, *Trichuris ovis*, *Pearsonema plica*
Viruses	Ebola virus (EBOV), Sudan virus (SUDV), Taï Forest virus (TAFV), Bundibugyo virus (BDBV), Lloviu virus (LLOV), Reston virus (RESTV) and Marburg virus (MARV), Zika virus, Group A rotaviruses (RVA), Leopards hill virus (LPHV), Adenoviruses, Paramyxoviruses, Orthoreovirus, Luna virus (LUAV), Lunk virus (LNKV), Encephalomyocarditis virus (EMCV), Polyomavirus (PyV), Orthopoxvirus, Hantavirus, and Pteropine orthoreovirus. Other viruses were Orthopox virus (OPXV), Polyomavirus (PyV), Bufavirus (BuV), Smacovirus (SmVs), Simian Immunodeficiency virus (SIV), Simian pegivirus (SPgV), and Simian arterivirus.

**Table 2:** Viral, bacterial, and protozoan pathogens reported in bats in Zambia, 1990 to 2022.

Pathogen	Bat species sampled	Reported and known public health risks	Sampling year	Location of sampling (District)	Prevalence [Sero-prevalence]	Ref
Filoviruses	*E. helvum*	EBOV, SUDV, and BDBV cause hemorrhagic fevers in humans	2006–2013	Ndola, Serenje	8.6% (64/748)	(37, 111)
	*R. aegyptiacus*		2014–2017	Kafue, Chongwe	10.7% (31/290)	(38)
Filoviruses (Orthomarburgviruses)	*E. helvum*	MARV causes hemorrhagic fevers in humans	2006–2013	Ndola, Serenje	[0.9% (7/748)]	(37)
	*R. aegyptiacus*	Close identity to MARV that caused human outbreaks in DRC	2014–2017	Chongwe	2.8% (2/71)	(43)
			2018			
RVA	*R. aegyptiacus*	Unknown	2014–15	Lusaka	5% (1/20)	(44)
	*E. helvum*	Unknown	2014–15	Ndola, Serenje^,^	10% (2/20)	(44)
	*Rhinolophus simulator*	98.1% nucleotide identity to a rotavirus from a sick child in Italy	2012–14	Chongwe	33.3% (1/3)	(34)
LPHV	*H. gigas*	Causes a haemorrhagic disease in mice similar to CCHFV in humans	2010–2012	Chongwe	27.1% (16/59)	(40)
Polyomavirus	*Rhinolophus* sp.	Unknown	2012–13	Chongwe	22.9% (8/35)	(112, 113)
	*M. schreibersi*	Unknown	2012–13		30.8% (3/13)	
Adenovirus	*E. helvum*	Unknown	2006,10–13	Serenje, Ndola	1.9% (9/472)	(45)
Paramyxovirus	*E. helvum*	Unknown	2008–2011	Serenje	8% (25/312)	(39)
Orthoreovirus	*R. aegyptiacus*	Unknown	2014–2015	Chongwe	1% (1/96)	(46)
Pteropine Orthoreovirus	*R. aegyptiacus*	Unknown	2018	Chongwe	2.1% (1/47)	(47)
					[97.3% (36/37)]	
	*E. helvum*	Unknown	2017, 2018	Ndola	[100% (33/33)]	
*Leptospira* sp.	*E. helvum*	Close identity to pathogenic *L. borgpetersenii* and *L. kirschneri*	2008–2013	Serenje, Ndola	14.9% (79/529)	(35)
*Ca. B. rousetti*	*R. aegyptiacus*	Close nucleotide similarity to *Ca. B.* rousetti whose antibodies have been reported in humans	2017–2018	Chongwe	16.7% (3/18)	(41)
	*Macronycteris vittatus*		2017–2018	Chongwe	16.7% (3/18)	
*B. faini*	*R. aegyptiacus*	Caused febrile illness in a human following a bite from a *Borrelia faini* positive tick (*Ornithodoros faini*) in a cave	2012–2013	Chongwe	33.3% (59/177)	(36)
	*Hipposideros* sp.				6.4% (3/47)	
	*Miniopterus* sp.				16.7% (2/12)	
*Trypanosoma* sp.	*Hipposideros vittatus*	Unknown	2017	Ndola, Serenje	11.6% (5/43)	(42)

**Table 3:** Viral and bacterial pathogens reported in rodents in Zambia, 1990 to 2022.

Pathogen	Host	Sampling year	Reported and known public health risks	Location of sampling (District)	Prevalence [Sero-prevalence]	Ref
Paramyxovirus	*M. natalensis*	2010–12	Unknown	Lusaka, Livingstone, Mpulungu	19.5% (84/431)	(31)
LUAV	*M. natalensis*	2009–11, 2021–22		Kafue, Livingstone, Lusaka, Namwala	9.6% (37/387)	(51, 52, 56)
LNKV	*M. minutoides*	2010–11	A variant of pathogenic Lymphocytic choriomeningitis virus	Lusaka	0.3% (1/3)	(52)
EMCV	*M. natalensis*	2012–13	Causes febrile illness in humans	Mpulungu, Solwezi	14% (19/136)	(55)
Polyomavirus	*Mastomys* sp.	2011	Unknown	Namwala	2.2% (1/45)	(50)
Orthopoxvirus	*M. natalensis*	2012–13		Solwezi, Mpulungu, Mazabuka	[15% (38/254)]	(33)
Hantavirus	*M. natalensis*	2006–10	Cause life-threatening cardiac, pulmonary and renal illness	Namwala & Lusaka	[1.8% (2/112)]	(49)
	*Steatomys* sp.	2006–10		Namwala	[2.7% (1/37)]	
	*Gerbillinae* sp.	2006–10		Namwala	[1.1% (1/92)]	
*Leptospira* sp.	*R. tanezumi*	2006–07	Unknown since only seroprevalence was reported	Lusaka	[5.9% (1/17)]	(49)
*Yersinia pestis*	*M. natalensis*	2006–10	Causes sporadic outbreaks of bubonic plague in humans in Zambia	Lusaka	[0.9% (1/112)]	(54)
		2016–17		Nyimba and Sinda	5.8% (19/329)	(23)
	*R. rattus*	2006–10		Namwala,	[7.7% (1/13)]	(54)
*Rickettsia felis*	*Mastomys* sp.	2015	Causes zoonotic flea-borne spotted fever in humans (114)	Lusaka, Namwala	11.3% (12/106)	(53)
*Coxiella burnetii*	*M. natalensis*	2012–13	Causes febrile illness and pneumonia in humans	Nyimba & Namwala	27.3% (3/11)	(48)
	*Gerbillinae* sp.				71.4% (5/7)	
	*S. campestris*				100% (2/2)	
*H. microstoma*	*M. natalensis*		Suspected zoonoses: reported in humans in the early 2000s	Kafue	3.8% (7/182)	(56)
*C. hepaticum*,	*M. natalensis*		hepatic capillariasis	Lusaka	0.5% (1/182)	(56)
*Trichnella spiralis*	*M. natalensis*		Trichinellosis	Kafue	0.5% (1/182)	
*Trichuris ovis*	*M. natalensis*		No evidence of human infections	Kafue	0.5% (1/182)	

**Table 4:** Viral, bacterial, and protozoan pathogens reported in NHPs in Zambia, 1990 to 2022.

Pathogen	NHP species	Sampling year	Reported and known public health risks	Location of sampling	Prevalence [Sero-prevalence]	Ref
*Rickettsia africae*	*P. cynocephalus*	2008	Causes Africa tick-bite fever	Mfuwe	33.3% (16/48)	(57)
	*C. pygerythrus*				47.5% (19/40)	
*A. phagocytophilum*	*P. cynocephalus*	2008	Causes human granulocytic anaplasmosis	Mfuwe	10.4% (5/48)	
	*C. pygerythrus*				17.5% (7/40)	
Simian Pegivirus,	*C. cynosures*	–	Unknown	KNP/Mumbwa	Not defined	(63)
Simian Arterivirus			Unknown		Not defined	
SIV	*C. cynosures*	2008–9	Novel species but unknown impact on humans	Mfuwe	3.2% (3/94)	(58)
Smacovirus	*C. cynosures*	2009	Unknown	Mfuwe	16% (4/25)	(64)
	*P. cynocephalus*	2009	Unknown		20% (4/20)	
	*P. kindae*	2009	Unknown		40% (2/5)	
Polyomavirus	*P. cynocephalus*	2009	Unknown	Mfuwe	35 (3/100)	(62)
	*C. pygerythrus*		Unknown		8% (4/50)	
Bufavirus	*P. cynocephalus*	2009	Unknown	Mfuwe	4% (2/50)	(32)
	*P. ursinus*	2010–11	Unknown	Livingstone	2% (1/50)	
Orthopoxvirus	*P. ursinus*	2009–11	Unknown		[2.1% (4/188)]	(33)
Zika virus	*P. ursinus,*	2009–10	Known to cause congenital abnormalities	Livingstone	[48% (12/25)]	(61)
	*C. cynosures*	2009–10		Mfuwe	[33.3% (16/48)]	
	*P. cynocephalus*	2009–10		Mfuwe	[21.7% (5/23)]	
Filoviruses (EBOV, BDBV, MARV, SUDV)	*Papio* sp., *Chlorocebus* sp.	2008–2010	Cause hemorrhagic fevers in humans	Mfuwe	[16% (39/243)]	(59)
				Livingstone		
HPIV3	*P. cynocephalus*	2009	Sequenced related to HPIV3 detected in a sick child in Saudi Arabia	Mfuwe	2% (1/50)	(60)
	*P. ursinus*	2010–11		Livingstone	6% (3/50)	
*T.b. rhodesiense*	*C. pygerythrus*	2020	Known to causes Human African Trypanosomiasis	KNP	100% (1/1)	(65)

### 3.6 Distribution and zoonotic potential of reported infectious agents by animal type

The 18 infectious agents reported in bats (Table 1) were primarily in *Rousettus aegyptiacus* and *Eidolon helvum*, comprising 77.8% (14/18) viruses, 16.7% (3/18) bacteria, and 5.5% (1/18) parasites. Noteworthy zoonotic viruses included EBOV, MARV, RVA, *Borrelia faini*, *Leptospira* sp., among others. In rodents, viruses and parasites each contributed 38.9% (7/18) to the reported infectious agents compared to 22.2% (4/18) by bacteria (Table 2). Most pathogens were reported in *Mastomys natalensis*. Notable pathogens were *Yersinia pestis*, *Rickettsia felis*, *Coxiella burnetti*, *Calodium hepaticum*, and *Leptospira* sp. Among NHPs, 71.4% (10/14) of the reported infectious agents were viruses (Table 3). Bacteria and parasites each accounted for 14.3% (2/14). Human parainfluenza virus type 3 (HPIV3), filoviruses, *Trypanosoma brucei rhodesiens,* and *Anaplasma phagocytophilum* were the headline pathogens.

## 4 Discussion

This review uncovered genetic and serological evidence of a worrying array of infectious agents, some with documented history of causing severe human illnesses in Zambia (23, 36, 66) and elsewhere (67–69). Among these were EBOV, SUDV, BDBV, MARV, RVA, ZIKV, HPIV3, EMCV, Hantavirus, *Leptospira* sp., *Ca. B. rousetti*, *B. faini*, *Y. pestis*, *R. felis*, *C. burnetti*, *A. phagocytophilum*, and *T. b. rhodesiense* (65, 67, 70–83). Three pathogens, *Y. pestis* (84), *B. faini* (36), and *T. b. rhodesiense* were detected in active human clinical illness alongside their presence in wildlife, highlighting the immediate threat these pathogens pose. Furthermore, LPHV and LNKV (40, 52) were flagged as public health concerns due their similarity in clinical symptoms observed in humans and animals (85), and their close genetic relationships with known highly pathogenic organisms (86), respectively. These findings emphasise the critical need for enhanced surveillance and comprehensive public health strategies to prevent and control potential outbreaks.

### 4.1 Quality of reviewed articles and research trends

All the articles included in the review utilized standard polymerase chain reaction (PCR) detection methods and reported the prevalence of pathogens, except for one study that did not (63). Sampling of non-human primates (NHPs) was predominantly focused on national parks in the Livingstone and Mfuwe districts, where the researchers had active projects. Additionally, the studies exhibited a bias toward viral pathogens, reflecting the specific interests of the researchers. Articles detailing zoonotic pathogen circulation in the animals of interest from 1990 to 2009 were absent. Although the reason for the absence of articles during that period could be linked to various factors, data from some review articles suggest a possible focus on anthrax, bovine tuberculosis, trypanosomiasis, tick-borne parasitic diseases, and other zoonoses in livestock (87–91). Therefore, there is need for further investigations over the observed trends in order to fully understand the evolution of research on zoonoses in Zambia. Nonetheless, post-2009, there was a notable surge in research involving bats, rodents, and NHPs (31–33, 35, 37, 39, 40, 49–52, 57, 60, 62). This increase in research activity likely stems from significant events such as the emergence of LUJV in Zambia in 2008 (1), the global swine influenza outbreak in 2009 (92), and the recurring, deadly outbreaks of Marburg virus disease (MDV) (70), and EVD in the neighbouring DRC (82).

### 4.2 Sampling approaches in the reviewed articles

Cross-sectional study designs were the most predominant, although they lacked subsequent follow-up research, except for two studies (37, 38). Therefore, the temporal dynamics related to abundance and likelihood of outbreaks of most reported pathogens remain poorly studied. These glaring gaps emphasise the critical need for robust long term surveillance studies in order to fully understand the spatial, temporal, and transmission dynamics of infectious agents within their micro-environments (56).

### 4.3 Organ-specific distribution of pathogens

Understanding organ-specific pathogen distribution enhances targeted surveillance and diagnostic strategies, crucial for predicting and controlling infectious diseases. Most studies focused on spleen, liver, kidneys, and blood, with one exception using semen samples (56), highlighting their potential for pathogen detection, including viruses like EBOV and LASV (56, 93–95). This underscores the need to screen multiple organs and fluids to increase detection rates. For filovirus surveillance in bats, semen and seminal vesicles may offer valuable insights, as traditional samples (blood, liver, etc.) have scarcely yielded positive RNA results (14, 19). Integrating diverse sample types could enhance disease surveillance and response efforts significantly.

### 4.4 Geographical distribution of reported pathogens

The distribution of pathogens mirrored the spread of their hosts. However, reservoir presence did not always correlate with infectious agents (51, 52, 56). Studies covered just 14 of 116 districts, notably neglecting Western and Luapula provinces with rich river systems ideal for diverse pathogens (56). This gap raises concerns about potential undetected zoonotic hotspots. Sparse surveillance hampered understanding of pathogen spread, crucial for guiding presumptive treatment in areas lacking multi-pathogen diagnostic capabilities in healthcare settings.

### 4.5 Public health concerns of reported viral pathogens

Serological and molecular evidence of bats and NHPs encountering pathogenic filoviruses raises significant public health concerns. Serological findings are notable as EBOV RNA has only been detected once in bats (19). EBOV and MARV cause deadly, unpredictable epidemics in Central and West Africa, with the 2014–2015 West African EBOV outbreak causing over 11,310 deaths (73, 96). In Zambia, spill-over risk stems from *R. aegyptiacus* bats, and migratory *E. helvum* bats from filovirus-endemic Congo Basin countries (97). Infected humans crossing the open border from the EBOV hotspot, DRC, are potential sources of human to human transmission (82). Wild RVAs (34, 44) also raise public health worries due to their potential impact on current vaccines (98), particularly the possible emergence of reassorted viruses not covered by existing vaccines (99). The discovery of an RVA with 98.1% nucleotide similarity to shows the potential cross species transmission between bats and humans probably through contaminated shared fruits and water (34). On the other hand, HPIV3, Hantavirus, Zika virus, and novel pathogens such as LPHV and LNKV cannot be ignored. Discovery of an HPIV3 sequence in baboons, closely resembling a strain isolated from a sick child in Saudi Arabia (60, 100), underscores the need for investigations into zoonotic HPIV3 in children in Zambia. Therefore, both known and novel viruses reported herein, demand rigorous investigation to mitigate their potential threat to local and global health.

### 4.6 Public health concerns of reported bacterial pathogens

*Yersinia pestis*, *C. burnetii*, *A. phagocytophilum*, and *Leptospira* sp. were the headline pathogens in the reviewed articles. *Yersinia pestis* poses a documented threat in Zambia, causing sporadic outbreaks of bubonic plague, particularly in eastern regions (23, 54). On the other hand, *C. burnetii*, responsible for Q fever which has never been reported in humans in Zambia, seems widespread within multiple rodent hosts in diverse ecological settings (101). *Leptospira* sp., similar to other zoonotic pathogens, suffers from a significant paucity of molecular and human data in Zambia. This calls for public health vigilance due to recent outbreaks in humans in neighbouring Tanzania (102). *Anaplasma phagocytophilum* was another significant zoonotic pathogen reported in wildlife. It is a multi-host intracellular pathogen, which causes varying severity of human febrile granulocytic anaplasmosis and encephalitis (103, 104). Like the emergence of *B. faini* which caused Zambia’s first clinical case of human borreliosis (36), its detection is alarming and warrants further investigations. The disease caused by *B. faini* presented with symptoms resembling malaria and flu, including high fever, initially confusing diagnosis at a local clinic, a common phenomenon with zoonotic pathogens (105). *B. faini* formed a monophyletic lineage akin to relapsing fever borreliae in the USA (36). Further investigations are critically needed to understand its overall impact on human health in Zambia.

### 4.7 Public health concerns of reported parasitic pathogens

Significant public health threats from pathogens including *T. b. rhodesiense*, *C. hepaticum*, *T. spiralis*, and *T. ovis* still exist as reported in the articles. Human African trypanosomiasis (HAT) caused by *T. b. rhodesiense* provided a problematic diagnostic process among clinicians, resulting in delayed treatment, suggesting need for refresher courses on this neglected zoonoses (65, 66). Strategic awareness campaigns may be helpful to educate the public about HAT and pathogenic parasites such as *C. hepaticum*, *T. spiralis*, and *T. ovis* (106–110).

### 4.8 Implication for the Southern Africa region and beyond

In the context of Zambia’s open boarders and its role as a key transportation hub for Southern Africa, the implications of the existence of highly pathogenic zoonotic organisms with potential for major outbreaks are even alarming. It significantly heightens the risk of zoonotic pathogen outbreaks spreading rapidly across the region. The free international connectivity and high traffic can swiftly transform a local outbreak into a pandemic. Economically, the region could suffer from costly public health crises, disrupting trade and travel, and impacting regional stability similar to COVID-19 (1). To mitigate the risk of zoonotic disease spread through these transportation hubs, robust screening and surveillance systems are vital. Continuous and consistent aggregation and analysis of available data on circulating zoonotic pathogens can help map hotspots and enhance the country and region’s ability to respond effectively.

## 5 Conclusion and future perspectives

The findings underscore Zambia’s critical public health challenge: diverse pathogens with zoonotic potential identified in bats, rodents, and non-human primates across a few districts. With only 14 out of 116 districts reporting data, much of the country’s wildlife pathogen landscape remains unexplored. Studies from which the data was extracted were characterised by short-term investigations lacking follow-up investigations and detailed analysis of the micro-characteristics of host habitats. This gap leaves the ecology of most reported pathogens poorly understood. To address this, future studies should comprehensively examine all aspects of pathogen prevalence, transmission, and persistence dynamics to identify potential hotspots, both spatially and temporally. Additionally, there is a huge gulf between what is known about zoonotic pathogens in wildlife and in humans in Zambia. Thus, comprehensive serological and molecular studies are urgently needed to reveal the true burden of these pathogens on human health to guide laboratory diagnosis and region-specific treatment options.

## Data Availability

The original contributions presented in the study are included in the article/Supplementary material, further inquiries can be directed to the corresponding author.

## References

[1] Simulundu E Mweene AS Changula K Monze M Chizema E Mwaba P . Lujo viral hemorrhagic fever: considering diagnostic capacity and preparedness in the wake of recent Ebola and Zika virus outbreaks. Rev Med Virol. (2016) 26:446–54. doi: 10.1002/rmv.1903, PMID: 27593704 PMC7169100

[2] Allen T Murray KA Zambrana-Torrelio C Morse SS Rondinini C Di Marco M . Global hotspots and correlates of emerging zoonotic diseases. Nat Commun. (2017) 8:1124. doi: 10.1038/s41467-017-00923-8, PMID: 29066781 PMC5654761

[3] Allocati N Petrucci AG Di Giovanni P Masulli M Di Ilio C De Laurenzi V. Bat–man disease transmission: zoonotic pathogens from wildlife reservoirs to human populations. Cell Death Discov. (2016) 2:16048. doi: 10.1038/cddiscovery.2016.48, PMID: 27551536 PMC4979447

[4] Delia G Mutua F Ochungo P Kruska R Jones K Brierley L . Mapping of poverty and likely zoonoses hotspots International Livestock Research Institute – Zoonoses Project. Nairobi, Kenya. (2012).

[5] Carlson CJ Zipfel CM Garnier R Bansal S. Global estimates of mammalian viral diversity accounting for host sharing. Nat Ecol Evol. (2019) 3:1070–5. doi: 10.1038/s41559-019-0910-6 31182813

[6] Carlson CJ Albery GF Merow C Trisos CH Zipfel CM Eskew EA . Climate change increases cross-species viral transmission risk. Nature. (2022) 607:555–62. doi: 10.1038/s41586-022-04788-w, PMID: 35483403

[7] Smith KM Machalaba CC Seifman R Feferholtz Y Karesh WB. Infectious disease and economics: the case for considering multi-sectoral impacts. One Health. (2019) 7:100080. doi: 10.1016/j.onehlt.2018.100080, PMID: 30671528 PMC6330263

[8] Fichet-Calvet E Lecompte E Koivogui L Soropogui B Doré A Kourouma F . Fluctuation of abundance and Lassa virus prevalence in *Mastomys natalensis* in Guinea, West Africa. Vector Borne Zoonotic Dis. (2007) 7:119–28. doi: 10.1089/vbz.2006.0520, PMID: 17627428

[9] Luby SP Rahman M Hossain MJ Blum LS Husain MM Gurley E . Foodborne transmission of Nipah virus, Bangladesh. Emerg Infect Dis. (2006) 12:1888–94. doi: 10.3201/eid1212.060732, PMID: 17326940 PMC3291367

[10] Subramanian M. Zoonotic disease risk and the Bushmeat trade: assessing awareness among hunters and traders in Sierra Leone. EcoHealth. (2012) 9:471–82. doi: 10.1007/s10393-012-0807-1, PMID: 23408099

[11] Islam MS Sazzad HMS Satter SM Sultana S Hossain MJ Hasan M . Nipah virus transmission from bats to humans associated with drinking traditional liquor made from date palm sap, Bangladesh, 2011-2014. Emerg Infect Dis. (2016) 22:664–70. doi: 10.3201/eid2204.151747, PMID: 26981928 PMC4806957

[12] Dahmana H Granjon L Diagne C Davoust B Fenollar F Mediannikov O. Rodents as hosts of pathogens and related zoonotic disease risk. Pathogens. (2020) 9:202. doi: 10.3390/pathogens9030202 32164206 PMC7157691

[13] Dietrich M Mühldorfer K Tortosa P Markotter W. Leptospira and bats: story of an emerging friendship. PLoS Pathog. (2015) 11:e1005176–6. doi: 10.1371/journal.ppat.1005176, PMID: 26562435 PMC4643053

[14] Koch LK Cunze S Kochmann J Klimpel S. Bats as putative Zaire ebolavirus reservoir hosts and their habitat suitability in Africa. Sci Rep. (2020) 10:14268. doi: 10.1038/s41598-020-71226-0, PMID: 32868789 PMC7459104

[15] Robertson SN Cameron AI Morales PR Burnside WM. West Nile virus Seroprevalence in an outdoor nonhuman primate breeding Colony in South Florida. J Am Assoc Lab Anim Sci. (2021) 60:168–75. doi: 10.30802/AALAS-JAALAS-20-000029, PMID: 33441221 PMC7974820

[16] Ayivor JS Ohemeng F Tweneboah Lawson E Waldman L Leach M Ntiamoa-Baidu Y. Living with bats: the case of Ve Golokuati township in the Volta region of Ghana. J Environ Public Health. (2017) 2017:5938934–11. doi: 10.1155/2017/5938934, PMID: 29081813 PMC5610796

[17] Epstein JH Anthony SJ Islam A Kilpatrick AM Ali Khan S Balkey MD . Nipah virus dynamics in bats and implications for spillover to humans. Proc Natl Acad Sci USA. (2020) 117:29190–201. doi: 10.1073/pnas.2000429117, PMID: 33139552 PMC7682340

[18] Amman BR Bird BH Bakarr IA Bangura J Schuh AJ Johnny J . Isolation of Angola-like Marburg virus from Egyptian rousette bats from West Africa. Nat Commun. (2020) 11:510. doi: 10.1038/s41467-020-14327-8, PMID: 31980636 PMC6981187

[19] Leroy EM Kumulungui B Pourrut X Rouquet P Hassanin A Yaba P . Fruit bats as reservoirs of Ebola virus. Nature. (2005) 438:575–6. doi: 10.1038/438575a, PMID: 16319873

[20] Delaune D Hul V Karlsson EA Hassanin A Ou TP Baidaliuk A . A novel SARS-CoV-2 related coronavirus in bats from Cambodia. Nat Commun. (2021) 12:6563. doi: 10.1038/s41467-021-26809-4, PMID: 34753934 PMC8578604

[21] Sun Y Lin W Dong W Xu J. Origin and evolutionary analysis of the SARS-CoV-2 omicron variant. J Biosaf Biosecur. (2022) 4:33–7. doi: 10.1016/j.jobb.2021.12.001, PMID: 35005525 PMC8718870

[22] Akhuemokhan OC Ewah-Odiase RO Akpede N Ehimuan J Adomeh DI Odia I . Prevalence of Lassa virus disease (LVD) in Nigerian children with fever or fever and convulsions in an endemic area. PLoS Negl Trop Dis. (2017) 11:e0005711. doi: 10.1371/journal.pntd.0005711, PMID: 28671959 PMC5510890

[23] Nyirenda SS Hang’ombe BM Simulundu E Mulenga E Moonga L Machang’u RS . Molecular epidemiological investigations of plague in Eastern Province of Zambia. BMC Microbiol. (2018) 18:2. doi: 10.1186/s12866-017-1146-8, PMID: 29433443 PMC5810007

[24] CDC. Diseases Spread by Rodents | Rodents | CDC. (2022). Available at: https://www.cdc.gov/rodents/diseases/index.html (accessed September 13, 2022).

[25] Gao F Bailes E Robertson DL Chen Y Rodenburg CM Michael SF . Origin of HIV-1 in the chimpanzee *Pan troglodytes troglodytes*. Nature. (1999) 397:436–41. doi: 10.1038/17130 9989410

[26] Khabbaz RF Heneine W George JR Parekh B Rowe T Woods T . Infection of a laboratory worker with simian immunodeficiency virus. N Engl J Med. (1994) 330:172–7. doi: 10.1056/NEJM199401203300304, PMID: 8264739

[27] Davies TJ Pedersen AB. Phylogeny and geography predict pathogen community similarity in wild primates and humans. Proc R Soc B Biol Sci. (2008) 275:1695–701. doi: 10.1098/rspb.2008.0284, PMID: 18445561 PMC2602822

[28] Devaux CA Mediannikov O Medkour H Raoult D. Infectious disease risk across the growing human-non human primate Interface: a review of the evidence. Front Public Health. (2019) 7:305. doi: 10.3389/fpubh.2019.00305, PMID: 31828053 PMC6849485

[29] Munjita SM Chileshe M Mutemwa S. Ebola virus disease in West Africa: a call to overhaul health systems in sub-Saharan Africa. Int J Med Sci Public Health. (2015) 4:873–3. doi: 10.5455/ijmsph.2015.03022015216

[30] fJoanna Briggs Institute. Critical appraisal checklist for prevalence studies. JBI (2020). Available at: https://jbi.global/about-jbi (accessed September 13, 2022).

[31] Sasaki M Muleya W Ishii A Orba Y Hang’ombe BM Mweene AS . Molecular epidemiology of paramyxoviruses in Zambian wild rodents and shrews. J Gen Virol. (2014) 95:325–30. doi: 10.1099/vir.0.058404-0, PMID: 24189618

[32] Sasaki M Orba Y Anindita P Ishii A Ueno K Hang’ombe B . Distinct lineages of Bufavirus in wild shrews and nonhuman Primates. Emerg Infect Dis J. (2015) 21:1230–3. doi: 10.3201/eid2107.141969 PMC4480391 26079728

[33] Orba Y Sasaki M Yamaguchi H Ishii A Thomas Y Ogawa H . Orthopoxvirus infection among wildlife in Zambia. J Gen Virol. (2015) 96:390–4. doi: 10.1099/vir.0.070219-0, PMID: 25319753

[34] Sasaki M Orba Y Sasaki S Gonzalez G Ishii A Hang’ombe BM . Multi-reassortant G3P[3] group a rotavirus in a horseshoe bat in Zambia. J Gen Virol. (2016) 97:2488–93. doi: 10.1099/jgv.0.000591, PMID: 27574104

[35] Ogawa H Koizumi N Ohnuma A Mutemwa A Hang’ombe BM Mweene AS . Molecular epidemiology of pathogenic Leptospira spp. in the straw-colored fruit bat (*Eidolon helvum*) migrating to Zambia from the Democratic Republic of Congo. Infect Genet Evol. (2015) 32:143–7. doi: 10.1016/j.meegid.2015.03.013, PMID: 25791930 PMC7106174

[36] Qiu Y Nakao R Hang’ombe BM Sato K Kajihara M Kanchela S . Human Borreliosis caused by a New World relapsing fever Borrelia–like organism in the Old World. Clin Infect Dis. (2019) 69:107–12. doi: 10.1093/cid/ciy850, PMID: 30423022

[37] Ogawa H Miyamoto H Nakayama E Yoshida R Nakamura I Sawa H . Seroepidemiological prevalence of multiple species of filoviruses in fruit bats (*Eidolon helvum*) migrating in Africa. J Infect Dis. (2015) 212:S101–8. doi: 10.1093/infdis/jiv063, PMID: 25786916

[38] Changula K Kajihara M Mori-Kajihara A Eto Y Miyamoto H Yoshida R . Seroprevalence of filovirus infection of *Rousettus aegyptiacus* bats in Zambia. J Infect Dis. (2018) 218:S312–7. doi: 10.1093/infdis/jiy266, PMID: 29889270

[39] Muleya W Sasaki M Orba Y Ishii A Thomas Y Nakagawa E . Molecular epidemiology of paramyxoviruses in frugivorous *Eidolon helvum* bats in Zambia. J Vet Med Sci. (2014) 76:611–4. doi: 10.1292/jvms.13-0518, PMID: 24389743 PMC4064153

[40] Ishii A Ueno K Orba Y Sasaki M Moonga L Hang’ombe BM . A nairovirus isolated from African bats causes haemorrhagic gastroenteritis and severe hepatic disease in mice. Nat Commun. (2014) 5:5651. doi: 10.1038/ncomms6651, PMID: 25451856 PMC4268697

[41] Qiu Y Kajihara M Nakao R Mulenga E Harima H Hang’ombe BM . Isolation of Candidatus Bartonella rousetti and other bat-associated Bartonellae from bats and their flies in Zambia. Pathogens. (2020) 9:469. doi: 10.3390/pathogens9060469, PMID: 32545824 PMC7350321

[42] Qiu Y Kajihara M Harima H Hang'ombe BM Nakao R Hayashida K . Molecular characterization and phylogenetic analysis of Trypanosoma spp. detected from striped leaf-nosed bats (*Hipposideros vittatus*) in Zambia. Int J Parasitol Parasites Wildl. (2019) 9:234–8. doi: 10.1016/j.ijppaw.2019.04.009, PMID: 31198682 PMC6555876

[43] Kajihara M Hang’ombe BM Changula K Harima H Isono M Okuya K . Marburgvirus in Egyptian Fruit Bats, Zambia. Emerg Infect Dis. (2019) 25:1577–80. doi: 10.3201/eid2508.190268, PMID: 31146800 PMC6649326

[44] Sasaki M Kajihara M Changula K Mori-Kajihara A Ogawa H Hang'ombe BM . Identification of group a rotaviruses from Zambian fruit bats provides evidence for long-distance dispersal events in Africa. Infect Genet Evol. (2018) 63:104–9. doi: 10.1016/j.meegid.2018.05.016, PMID: 29792990 PMC7173303

[45] Ogawa H Kajihara M Nao N Shigeno A Fujikura D Hang’ombe B . Characterization of a novel bat adenovirus isolated from straw-colored fruit bat (*Eidolon helvum*). Viruses. (2017) 9:371. doi: 10.3390/v9120371, PMID: 29207524 PMC5744146

[46] Harima H Sasaki M Kajihara M Mori-Kajihara A Hang’ombe BM Changula K . Detection of novel orthoreovirus genomes in shrew (*Crocidura hirta*) and fruit bat (*Rousettus aegyptiacus*). J Vet Med Sci. (2020) 82:162–7. doi: 10.1292/jvms.19-0424, PMID: 31866632 PMC7041985

[47] Harima H Sasaki M Orba Y Okuya K Qiu Y Wastika CE . Attenuated infection by a Pteropine orthoreovirus isolated from an Egyptian fruit bat in Zambia. PLoS Negl Trop Dis. (2021) 15:e0009768. doi: 10.1371/journal.pntd.0009768, PMID: 34492038 PMC8448348

[48] Chitanga S Simulundu E Simuunza MC Changula K Qiu Y Kajihara M . First molecular detection and genetic characterization of *Coxiella burnetii* in Zambian dogs and rodents. Parasit Vectors. (2018) 11:40. doi: 10.1186/s13071-018-2629-7, PMID: 29343277 PMC5773031

[49] Nakamura I Hang’ombe BM Sawa H Kobayashi S Orba Y Ishii A . Cross-reactivity of secondary antibodies against African rodents and application for sero-surveillance. J Vet Med Sci. (2013) 75:819–25. doi: 10.1292/jvms.12-0471, PMID: 23386359

[50] Orba Y Kobayashi S Nakamura I Ishii A Hang'ombe BM Mweene AS . Detection and characterization of a novel polyomavirus in wild rodents. J Gen Virol. (2011) 92:789–95. doi: 10.1099/vir.0.027854-0, PMID: 21177925

[51] Ishii A Thomas Y Moonga L Nakamura I Ohnuma A Hang’ombe B . Novel arenavirus, Zambia. Emerg Infect Dis. (2011) 17:1921–4. doi: 10.3201/eid1710.10452, PMID: 22000372 PMC3310648

[52] Ishii A Thomas Y Moonga L Nakamura I Ohnuma A Hang’ombe BM . Molecular surveillance and phylogenetic analysis of Old World arenaviruses in Zambia. J Gen Virol. (2012) 93:2247–51. doi: 10.1099/vir.0.044099-0, PMID: 22815269

[53] Moonga LC Hayashida K Nakao R Lisulo M Kaneko C Nakamura I . Molecular detection of *Rickettsia felis* in dogs, rodents and cat fleas in Zambia. Parasit Vectors. (2019) 12:168. doi: 10.1186/s13071-019-3435-6, PMID: 30975188 PMC6460736

[54] Nyirenda SS Hang’ombe BM Mulenga E Kilonzo BS. Serological and PCR investigation of *Yersinia pestis* in potential reservoir hosts from a plague outbreak focus in Zambia. BMC Res Notes. (2017) 10:345. doi: 10.1186/s13104-017-2667-9, PMID: 28754138 PMC5534097

[55] Kishimoto M Hang’ombe BM Hall WW Orba Y Sawa H Sasaki M. *Mastomys natalensis* is a possible natural rodent reservoir for encephalomyocarditis virus. J Gen Virol. (2021) 102:1564. doi: 10.1099/jgv.0.001564, PMID: 33533710

[56] Munjita SM Moonga G Mukubesa AN Ndebe J Mubemba B Vanaerschot M . Luna virus and helminths in wild *Mastomys natalensis* in two contrasting habitats in Zambia: risk factors and evidence of virus dissemination in semen. Pathogens. (2022) 11:1345. doi: 10.3390/pathogens11111345, PMID: 36422597 PMC9697851

[57] Nakayima J Hayashida K Nakao R Ishii A Ogawa H Nakamura I . Detection and characterization of zoonotic pathogens of free-ranging non-human primates from Zambia. Parasit Vectors. (2014) 7:490. doi: 10.1186/s13071-014-0490-x, PMID: 25358853 PMC4221724

[58] Carr M Kawaguchi A Sasaki M Gonzalez G Ito K Thomas Y . Isolation of a simian immunodeficiency virus from a malbrouck (*Chlorocebus cynosuros*). Arch Virol. (2017) 162:543–8. doi: 10.1007/s00705-016-3129-8, PMID: 27804019

[59] Changula K Simulundu E Lombe BP Nakayama E Miyamoto H Takahashi Y . Serological evidence of filovirus infection in nonhuman Primates in Zambia. Viruses. (2021) 13:1283. doi: 10.3390/v13071283 34209295 PMC8309988

[60] Sasaki M Ishii A Orba Y Thomas Y Hang'ombe B Moonga L . Human parainfluenza virus type 3 in wild nonhuman primates, Zambia. Emerg Infect Dis. (2013) 19:1500–3. doi: 10.3201/eid1909.121404, PMID: 23968816 PMC3810902

[61] Wastika CE Sasaki M Yoshii K Anindita PD Hang’ombe BM Mweene AS . Serological evidence of Zika virus infection in non-human primates in Zambia. Arch Virol. (2019) 164:2165–70. doi: 10.1007/s00705-019-04302-0, PMID: 31154511

[62] Yamaguchi H Kobayashi S Ishii A Ogawa H Nakamura I Moonga L . Identification of a novel polyomavirus from vervet monkeys in Zambia. J Gen Virol. (2013) 94:1357–64. doi: 10.1099/vir.0.050740-0, PMID: 23426354

[63] Bailey AL Lauck M Ghai RR Nelson CW Heimbruch K Hughes AL . Arteriviruses, Pegiviruses, and lentiviruses are common among wild African monkeys. J Virol. (2016) 90:6724–37. doi: 10.1128/JVI.00573-16, PMID: 27170760 PMC4944300

[64] Anindita PD Sasaki M Gonzalez G Phongphaew W Carr M Hang’ombe BM . Discovery and genetic characterization of diverse smacoviruses in Zambian non-human primates. Sci Rep. (2019) 9:5045. doi: 10.1038/s41598-019-41358-z, PMID: 30962460 PMC6453971

[65] Squarre D Hayashida K Gaithuma A Chambaro H Kawai N Moonga L . Diversity of trypanosomes in wildlife of the Kafue ecosystem, Zambia. Int J Parasitol. (2020) 12:34–41. doi: 10.1016/j.ijppaw.2020.04.005 PMC7215119 32420023

[66] Squarre D Kabongo I Munyeme M Mumba C Mwasinga W Hachaambwa L . Human African Trypanosomiasis in the Kafue National Park, Zambia. PLoS Negl Trop Dis. (2016) 10:e0004567. doi: 10.1371/journal.pntd.0004567 27196336 PMC4873190

[67] Ospina ML Tong VT Gonzalez M Valencia D Mercado M Gilboa SM . Zika virus disease and pregnancy outcomes in Colombia. N Engl J Med. (2020) 383:537–45. doi: 10.1056/NEJMoa1911023, PMID: 32757522 PMC7480270

[68] Marí Saéz A Weiss S Nowak K Lapeyre V Zimmermann F Düx A . Investigating the zoonotic origin of the west African Ebola epidemic. EMBO Mol Med. (2015) 7:17–23. doi: 10.15252/emmm.201404792, PMID: 25550396 PMC4309665

[69] Allan KJ Biggs HM Halliday JEB Kazwala RR Maro VP Cleaveland S . Epidemiology of leptospirosis in Africa: a systematic review of a neglected zoonosis and a paradigm for ‘one health’ in Africa. PLoS Negl Trop Dis. (2015) 9:e0003899. doi: 10.1371/journal.pntd.0003899 26368568 PMC4569256

[70] Towner JS Khristova ML Sealy TK Vincent MJ Erickson BR Bawiec DA . Marburgvirus genomics and association with a large hemorrhagic fever outbreak in Angola. J Virol. (2006) 80:6497–516. doi: 10.1128/JVI.00069-06, PMID: 16775337 PMC1488971

[71] Harbeck M Seifert L Hänsch S Wagner DM Birdsell D Parise KL . *Yersinia pestis* DNA from skeletal remains from the 6th century AD reveals insights into Justinianic plague. PLoS Pathog. (2013) 9:e1003349. doi: 10.1371/journal.ppat.1003349, PMID: 23658525 PMC3642051

[72] Lemtudo AP Mutai BK Mwamburi L Waitumbi JN. Seroprevalence of *Coxiella burnetii* in patients presenting with acute febrile illness at Marigat District hospital, Baringo County, Kenya. Vet Med Sci. (2021) 7:2093–9. doi: 10.1002/vms3.493 33955713 PMC8464244

[73] Kamorudeen RT Adedokun KA Olarinmoye AO. Ebola outbreak in West Africa, 2014 – 2016: epidemic timeline, differential diagnoses, determining factors, and lessons for future response. J Infect Public Health. (2020) 13:956–62. doi: 10.1016/j.jiph.2020.03.014 32475805

[74] Philip N Bahtiar Affendy N Ramli SNA Arif M Raja P Nagandran E . Leptospira interrogans and *Leptospira kirschneri* are the dominant Leptospira species causing human leptospirosis in Central Malaysia. PLoS Negl Trop Dis. (2020) 14:e0008197. doi: 10.1371/journal.pntd.0008197, PMID: 32203511 PMC7117766

[75] Bai Y Osinubi MOV Osikowicz L McKee C Vora NM Rizzo MR . Human exposure to novel Bartonella species from contact with fruit bats. Emerg Infect Dis. (2018) 24:2317–23. doi: 10.3201/eid2412.181204 30457529 PMC6256376

[76] Kingry LC Anacker M Pritt B Bjork J Respicio-Kingry L Liu G . Surveillance for and discovery of Borrelia species in US patients suspected of Tickborne illness. Clin Infect Dis. (2018) 66:1864–71. doi: 10.1093/cid/cix1107, PMID: 29272385 PMC5985202

[77] De Grazia S Martella V Giammanco GM Gòmara MI Ramirez S Cascio A . Canine-origin G3P[3] rotavirus strain in child with acute gastroenteritis. Emerg Infect Dis. (2007) 13:1091–3. doi: 10.3201/eid1307.070239, PMID: 18214189 PMC2878246

[78] Chua KB Voon K Crameri G Tan HS Rosli J McEachern JA . Identification and characterization of a new orthoreovirus from patients with acute respiratory infections. PLoS One. (2008) 3:e3803–3. doi: 10.1371/journal.pone.0003803, PMID: 19030226 PMC2583042

[79] Abdad MY Stenos J Graves S. *Rickettsia felis*, an emerging flea-transmitted human pathogen. Emerg Health Threats J. (2011) 4:7168–8. doi: 10.3402/ehtj.v4i0.7168, PMID: 24149035 PMC3168219

[80] Oberste MS Gotuzzo E Blair P Nix WA Ksiazek TG Comer JA . Human febrile illness caused by encephalomyocarditis virus infection, Peru. Emerg Infect Dis. (2009) 15:640–6. doi: 10.3201/eid1504.081428, PMID: 19331761 PMC2671410

[81] Zhang L Wang G Liu Q Chen C Li J Long B . Molecular analysis of *Anaplasma phagocytophilum* isolated from patients with febrile diseases of unknown etiology in China. PLoS One. (2013) 8:e57155. doi: 10.1371/journal.pone.0057155, PMID: 23451170 PMC3579781

[82] Claude KM Underschultz J Hawkes MT. Ebola virus epidemic in war-torn eastern DR Congo. Lancet. (2018) 392:1399–401. doi: 10.1016/S0140-6736(18)32419-X, PMID: 30297137

[83] Simusika P Bateman AC Theo A Kwenda G Mfula C Chentulo E . Identification of viral and bacterial pathogens from hospitalized children with severe acute respiratory illness in Lusaka, Zambia, 2011–2012: a cross-sectional study. BMC Infect Dis. (2015) 15:52. doi: 10.1186/s12879-015-0779-1, PMID: 25888024 PMC4391483

[84] McClean KL. An outbreak of plague in Northwestern Province, Zambia. Clin Infect Dis. (1995) 21:650–2. doi: 10.1093/clinids/21.3.650, PMID: 8527559

[85] Kajihara M Simuunza M Saasa N Dautu G Mori-Kajihara A Qiu Y . Serologic and molecular evidence for circulation of Crimean-Congo hemorrhagic fever virus in ticks and cattle in Zambia. PLoS Negl Trop Dis. (2021) 15:e0009452. doi: 10.1371/journal.pntd.0009452, PMID: 34061841 PMC8195391

[86] N' Dilimabaka N Berthet N Rougeron V Mangombi JB Durand P Maganga GD . Evidence of lymphocytic choriomeningitis virus (LCMV) in domestic mice in Gabon: risk of emergence of LCMV encephalitis in Central Africa. J Virol. (2015) 89:1456–60. doi: 10.1128/JVI.01009-14, PMID: 25378495 PMC4300659

[87] Makala LH Mangani P Fujisaki K Nagasawa H. The current status of major tick borne diseases in Zambia. Vet Res. (2003) 34:27–45. doi: 10.1051/vetres:2002056 12588682

[88] Simulundu E Lubaba CH van Heerden J Kajihara M Mataa L Chambaro HM . The epidemiology of African swine fever in “nonendemic” regions of Zambia (1989-2015): implications for disease prevention and control. Viruses. (2017) 9:236. doi: 10.3390/v9090236, PMID: 28832525 PMC5618003

[89] Siamudaala VM Bwalya JM Munang'andu HM Sinyangwe PG Banda F Mweene AS . Ecology and epidemiology of anthrax in cattle and humans in Zambia. Jpn J Vet Res. (2006) 54:15–23. PMID: 16786974

[90] Okabayashi T Hasebe F Samui KL Mweene AS Pandey SG Yanase T . Short report: prevalence of antibodies against spotted fever, murine typhus, and Q fever rickettsiae in humans living in Zambia. Am J Trop Med Hyg. (1999) 61:70–2. doi: 10.4269/ajtmh.1999.61.70, PMID: 10432059

[91] Mwanakasale V Songolo P. Disappearance of some human African trypanosomiasis transmission foci in Zambia in the absence of a tsetse fly and trypanosomiasis control program over a period of forty years. Trans R Soc Trop Med Hyg. (2011) 105:167–72. doi: 10.1016/j.trstmh.2010.12.002 21276598

[92] Gibbs AJ Armstrong JS Downie JC. From where did the 2009 “swine-origin” influenza a virus (H1N1) emerge? Virol J. (2009) 6:207. doi: 10.1186/1743-422X-6-207, PMID: 19930669 PMC2787513

[93] Thorson AE Deen GF Bernstein KT Liu WJ Yamba F Habib N . Persistence of Ebola virus in semen among Ebola virus disease survivors in Sierra Leone: a cohort study of frequency, duration, and risk factors. PLoS Med. (2021) 18:e1003273. doi: 10.1371/JOURNAL.PMED.1003273, PMID: 33566817 PMC7875361

[94] McElroy AK Akondy RS Harmon JR Ellebedy AH Cannon D Klena JD . A case of human Lassa virus infection with robust acute T-cell activation and Long-term virus-specific T-cell responses. J Infect Dis. (2017) 215:1862–72. doi: 10.1093/infdis/jix201, PMID: 28863472 PMC5853890

[95] Thielebein A Ighodalo Y Taju A Olokor T Omiunu R Esumeh R . Virus persistence after recovery from acute Lassa fever in Nigeria: a 2-year interim analysis of a prospective longitudinal cohort study. Lancet Microbe. (2022) 3:e32–40. doi: 10.1016/S2666-5247(21)00178-6, PMID: 35544114

[96] Okesanya OJ Manirambona E Olaleke NO Osumanu HA Faniyi AA Bouaddi O . Rise of Marburg virus in Africa: a call for global preparedness. Ann Med Surg. (2023) 85:5285–90. doi: 10.1097/MS9.0000000000001257 PMC10553126 37811021

[97] Ossa G Kramer-Schadt S Peel AJ Scharf AK Voigt CC. The movement ecology of the straw-colored fruit bat, *Eidolon helvum*, in sub-Saharan Africa assessed by stable isotope ratios. PLoS One. (2012) 7:e45729. doi: 10.1371/journal.pone.0045729, PMID: 23029206 PMC3448674

[98] Li K Lin X-D Huang K-Y Zhang B Shi M Guo W-P . Identification of novel and diverse rotaviruses in rodents and insectivores, and evidence of cross-species transmission into humans. Virology. (2016) 494:168–77. doi: 10.1016/j.virol.2016.04.017, PMID: 27115729 PMC7173014

[99] Simsek C Corman VM Everling HU Lukashev AN Rasche A Maganga GD . At least seven distinct rotavirus genotype constellations in bats with evidence of Reassortment and zoonotic transmissions. MBio. (2021) 12:e02755-20. doi: 10.1128/mbio.02755-20, PMID: 33468689 PMC7845630

[100] Almajhdi FN Alshaman MS Amer HM. Molecular characterization and phylogenetic analysis of human parainfluenza virus type 3 isolated from Saudi Arabia. J Med Virol. (2012) 84:1304–11. doi: 10.1002/jmv.23326, PMID: 22711360

[101] Vanderburg S Rubach MP Halliday JEB Cleaveland S Reddy EA Crump JA. Epidemiology of *Coxiella burnetii* infection in Africa: a OneHealth systematic review. PLoS Negl Trop Dis. (2014) 8:e2787. doi: 10.1371/journal.pntd.0002787, PMID: 24722554 PMC3983093

[102] Masunga DS Rai A Abbass M Uwishema O Wellington J Uweis L . Leptospirosis outbreak in Tanzania: an alarming situation. Ann Med Surg. (2022) 80:104347. doi: 10.1016/j.amsu.2022.104347, PMID: 35992205 PMC9382409

[103] Elhamiani Khatat S Sahibi H Hing M Alaoui Moustain I El Amri H Benajiba M . Human exposure to *Anaplasma phagocytophilum* in two cities of northwestern Morocco. PLoS One. (2016) 11:e0160880. doi: 10.1371/journal.pone.0160880, PMID: 27532208 PMC4988626

[104] Cosiquien RJS Stojiljkovic N Nordstrom CW Amadi E Lutwick L Dumic I. *Anaplasma phagocytophilum* encephalitis: a case report and literature review of neurologic manifestations of Anaplasmosis. Infect Dis Rep. (2023) 15:354–9. doi: 10.3390/idr15040035, PMID: 37489389 PMC10366838

[105] Zhang L Ma Q Zhang Y Sun B Zhao L. Analysis of misdiagnosed cases of hemorrhagic fever with renal syndrome in children: two cases and literature review. BMC Nephrol. (2019) 20:383. doi: 10.1186/s12882-019-1562-0, PMID: 31646967 PMC6813044

[106] Manor U Doviner V Kolodziejek J Weidinger P Dagan A Ben-Haim M . Capillaria hepatica (syn. Calodium hepaticum) as a cause of asymptomatic liver mass. Am J Trop Med Hyg. (2021) 105:204–6. doi: 10.4269/ajtmh.21-0120, PMID: 33999846 PMC8274773

[107] Mukaratirwa S La Grange L Pfukenyi DM. Trichinella infections in animals and humans in sub-Saharan Africa: a review. Acta Trop. (2013) 125:82–9. doi: 10.1016/j.actatropica.2012.09.005 23041114

[108] Summers RW Elliott DE Urban JF Jr Thompson R Weinstock JV. Trichuris suis therapy in Crohn’s disease. Gut. (2005) 54:87–90. doi: 10.1136/gut.2004.041749, PMID: 15591509 PMC1774382

[109] Gonçalves AQ Ascaso C Santos I Serra PT Julião GR Orlandi PP. Calodium hepaticum: household clustering transmission and the finding of a source of human spurious infection in a community of the amazon region. PLoS Negl Trop Dis. (2012) 6:e1943. doi: 10.1371/journal.pntd.0001943, PMID: 23285301 PMC3527340

[110] Fuehrer H-P. An overview of the host spectrum and distribution of Calodium hepaticum (syn. Capillaria hepatica): part 1-Muroidea. Parasitol Res. (2014) 113:619–40. doi: 10.1007/s00436-013-3691-x, PMID: 24248632 PMC3902076

[111] Munjita SM Kwenda G. Descriptive analysis of Ebola virus proteins: towards development of effective therapeutics and vaccines. Microbiol Res J Int. (2015) 8:457–79. doi: 10.9734/BMRJ/2015/16297

[112] Carr M Gonzalez G Sasaki M Dool SE Ito K Ishii A . Identification of the same polyomavirus species in different African horseshoe bat species is indicative of short-range host-switching events. J Gen Virol. (2017) 98:2771–85. doi: 10.1099/jgv.0.000935, PMID: 28984241

[113] Carr M Gonzalez G Sasaki M Ito K Ishii A Hang’ombe BM . Discovery of African bat polyomaviruses and infrequent recombination in the large T antigen in the Polyomaviridae. J Gen Virol. (2017) 98:726–38. doi: 10.1099/jgv.0.000737, PMID: 28430100

[114] Schriefer ME Sacci JB Dumler JS Bullen MG Azad AF. Identification of a novel rickettsial infection in a patient diagnosed with murine typhus. J Clin Microbiol. (1994) 32:949–54. doi: 10.1128/jcm.32.4.949-954.1994, PMID: 8027348 PMC267160

