# Supplementary material for: Unveiling the hidden threats: a review of pathogen diversity and public health risks from bats, rodents, and non-human primates in Zambia (1990–2022)
Source: Front Public Health. 2024 Nov 20;12:1471452. doi: 10.3389/fpubh.2024.1471452 (PMC11621629; doi:10.3389/fpubh.2024.1471452)
Supplement: Supplementary file 1 [file Table_1.docx]

| **Pathogen** | **Reservoir host** | **Potential Hotspot** | **Hotspot characteristics** |
| --- | --- | --- | --- |
| Marburg virus | *R. aegyptiacus* bats | Chongwe | Relatively hilly rural agricultural region featuring caves inhabited by bats |
| Group A rotavirus | *R. simulator* bats | Chongwe |  |
| Leopards hill virus | *H. gigas* bats | Chongwe |  |
| *Borrelia faini* | *R. aegyptiacus* bats | Chongwe |  |
|  | *Hipposideros* sp bats | Chongwe |  |
|  | *Miniopterus sp* bats | Chongwe |  |
| *Leptospira* sp. | *E. helvum* | Serenje | Rural farming region, includes a national park |
|  |  | Ndola | Features abundant tree cover, borders DRC |
| *Calodium hepaticum* | *Mastomys natalensis* | Lusaka (state lodge) | Hilly, preserved forests, and fallow land |
| Lunk virus | *Mus minutoides* |  |  |
| *Rickettsia felis* | *Mastomys natalensis* | Namwala | Rural agricultural area borders a flood plain and national park |
| *Coxiella burnetti* | *Mastomys natalensis*, *Gerbillinae* sp, *Saccostomus campestris* | Namwala |  |
|  |  | Nyimba | Rural agricultural areas where rodents are considered a delicacy |
| *Yersinia pestis* | *Mastomys natalensis* | Nyimba, Sinda |  |
| *Hymenolepis microstoma* | *Mastomys natalensis* | Kafue (Lower Zambezi) | Rural, part of the lower Zambezi basin and game management area |
| *Trichnella spiralis* |  |  |  |
| *Rickettsia africae* | *P. cynocephalus* | Mfuwe | Rural and includes the South Luangwa National Park |
|  | *C. pygerythrus* |  |  |
| *A. phagocytophilum* | *P. cynocephalus, C. pygerythrus* | Mfuwe |  |
| HPIV3 | *P. cynocephalus,* | Mfuwe |  |
|  | *P. ursinus* | Livingstone | Includes a waterfalls and a national park |
| *Trypanosoma brucei rhodesiense* | *C. pygerythrus* | Kafue national park | National park |
| Zika virus | *Papio ursinus, C. cynosures, P. cynocephalus* | Livingstone | As described earlier in the table |
|  |  | Mfuwe | As described earlier in the table |
| Hantavirus | Mastomys sp., Steatomys sp., Gerbillinae sp | Lusaka | As described earlier in the table |
|  |  | Namwala | As described earlier in the table |
| Ebola virus and related filoviruses (BDBV, SUDV) | *Papio* sp, *Chlorocebus* sp | Mfuwe, Livingstone | As described earlier in the table |
|  | *R. aegyptiacus, E. helvum* | Ndola, Serenje, Kafue, Chongwe | As described earlier in the table |

Table S1: Aggregated data of zoonotic pathogens in bats, rodents, and NHPs in Zambia
